# Predicting Bloodstream Infection in Pediatric Post-Transfusion Febrile Neutropenia: Development of a Simple Bedside Risk Score

**DOI:** 10.3390/children12121666

**Published:** 2025-12-08

**Authors:** Eui Jun Lee, Jae Yun Jung, Young Ho Kwak, Do Kyun Kim, Joong Wan Park

**Affiliations:** 1 Department of Emergency Medicine, Seoul National University College of Medicine, Seoul 03080, Republic of Koreazzibii17@snu.ac.kr (J.W.P.); 2 Department of Emergency Medicine, Seoul National University Hospital, Seoul 03080, Republic of Korea

**Keywords:** bloodstream infection, chemotherapy, febrile neutropenia, pediatrics, transfusion

## Abstract

Background/Objectives: Children receiving chemotherapy are highly susceptible to infection, and bloodstream infection (BSI) is a major cause of morbidity in febrile neutropenia. Post-transfusion fever represents a specific diagnostic dilemma, where febrile non-hemolytic transfusion reactions may be clinically indistinguishable from early BSI. We aimed to develop and internally validate a simple bedside score to predict BSI in children presenting to the ED with post-transfusion fever. Methods: We performed a retrospective, single-center diagnostic prediction study of consecutive ED encounters between 2015 and 2024 in a tertiary children’s hospital. Eligible encounters involved patients ≤ 18 years with an underlying malignancy receiving systemic chemotherapy who presented with fever within 24 h of red blood cell or platelet transfusion, had neutropenia, and with at least one blood culture obtained. BSI was defined as growth of a clinically significant pathogen within 48 h; episodes with only contaminants or colonizing flora were classified as non-BSI. Multivariable logistic regression with four prespecified predictors—transfusion-to-ED arrival interval, body temperature, absolute neutrophil count (ANC), and C-reactive protein (CRP)—was used to develop the model and derive a 0–5 point bedside score. Performance was assessed using AUC, diagnostic indices at prespecified cut-offs, calibration, and bootstrap internal validation. Results: Of 507 screened encounters, 287 met inclusion criteria; 39 (13.6%) were adjudicated as BSI. The full model showed good discrimination (AUC 0.82). The derived score (2 points for ANC = 0/µL; 1 point each for temperature ≥ 38.5 °C, CRP ≥ 2.5 mg/dL, and transfusion-to-ED interval ≥ 7 h) achieved an AUC of 0.84. At a cut-off ≥2, sensitivity was 97.4% and negative predictive value 98.8%, misclassifying 1 of 39 BSIs as low risk; at ≥3, specificity was 59.7% with sensitivity 89.7%. Bootstrap-corrected AUC was 0.83. Conclusions: In children receiving chemotherapy who present with post-transfusion fever, a simple 0–5 point bedside score based on temperature, ANC, CRP, and transfusion-to-ED interval provided useful early stratification of BSI risk in this single-center cohort. Prospective multicenter validation is needed before clinical implementation.

## 1. Introduction

Children undergoing chemotherapy for malignancies are profoundly susceptible to infections due to treatment-induced myelosuppression. Among these patients, bloodstream infections (BSI) represent a critical complication associated with substantial morbidity and mortality if not promptly identified and managed. Consequently, fever serves as a cardinal sign that necessitates immediate clinical evaluation and the initiation of empiric broad-spectrum antibiotics, which constitute the standard of care [1].

Alongside infection management, these children require extensive supportive care to mitigate treatment toxicity. Frequent transfusions of red blood cells (RBCs) and platelets are a cornerstone of this support for anemia and thrombocytopenia. However, transfusion itself acts as a confounding factor, being a well-established cause of non-infectious fever, most commonly febrile non-hemolytic transfusion reactions (FNHTRs). Differentiating these reactions from true infection presents a complex diagnostic challenge. Biologically, FNHTRs are often characterized by a rapid, passive inflammatory response to cytokines accumulated in the transfused product, whereas BSIs involve a slower, host-driven immune response to replicating pathogens. Despite these potential temporal and mechanistic differences, the clinical presentation—often high fever and rigors occurring during or shortly after transfusion—can be indistinguishable, making it difficult to rule out a life-threatening infection [2,3,4].

This diagnostic uncertainty is particularly problematic in the emergency department (ED), where rapid risk stratification is essential. It creates a dilemma of competing risks: misattributing an infectious fever to a transfusion reaction may delay crucial antimicrobial therapy, while presuming that infection in all cases contributes to antibiotic overuse, prolonged hospitalizations, and unnecessary expenditure of medical resources [5,6]. Accurate discrimination is therefore vital not only to ensure patient safety but also to optimize healthcare resource utilization. Although risk stratification tools exist for general febrile neutropenia (FN), there is a distinct lack of evidence-based guidance specifically for differentiating infectious from non-infectious etiologies in the specific niche of post-transfusion fever [1,7,8,9].

To address this clinical gap, we conducted a retrospective single-center diagnostic prediction study of post-transfusion febrile episodes in pediatric cancer patients. The primary objective was to identify key clinical and laboratory predictors distinguishing culture-confirmed BSIs from non-infectious fevers—with a specific focus on the validity of the transfusion-to-ED presentation interval. Based on these findings, we aimed to derive and internally validate a simple bedside risk score to facilitate rapid, evidence-based decision-making at the point of care.

## 2. Materials and Methods

### 2.1. Study Design and Population

**Figure 1:**
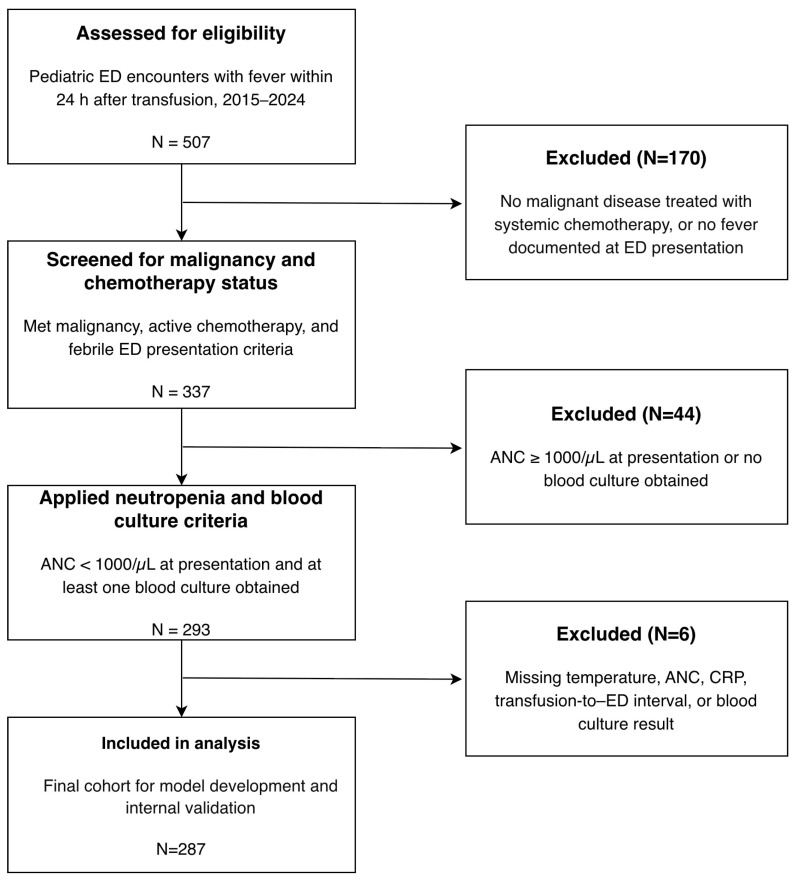
Study flow diagram.

This study was a retrospective, single-center diagnostic prediction study based on a consecutive cohort of pediatric ED encounters at a tertiary children’s hospital. We included all eligible encounters between 1 January 2015 and 31 December 2024. Eligible encounters met the following criteria: (1) an underlying malignant disease with the child actively receiving systemic chemotherapy; (2) presentation to the ED with fever occurring within 24 h of a documented red blood cell or platelet transfusion; and (3) documented neutropenia at or shortly after ED arrival, with at least one blood culture obtained to evaluate for bloodstream infection. Encounters were excluded if (1) the underlying diagnosis was not clearly a malignant disease treated with systemic chemotherapy; (2) the ED visit was not for a transfusion-related febrile neutropenia episode (e.g., no fever documented at presentation or during the initial ED assessment); or (3) essential investigations required for this study, such as the neutrophil count at presentation or blood culture results, were missing. The flow of patient selection and the numbers excluded at each step are summarized in Figure 1.

### 2.2. Definitions

The primary outcome was bloodstream infection. BSI was defined as the isolation of at least one clinically significant pathogenic organism from a blood culture obtained within 48 h of ED presentation in the setting of fever. Blood cultures were processed and interpreted according to standard institutional microbiology procedures. Organisms that were judged by the microbiology laboratory and the treating clinical team to represent contamination or colonizing flora were not considered evidence of BSI. Episodes in which blood cultures yielded only such organisms were adjudicated as contamination or colonization and were therefore classified as non-BSI in the primary analysis.

According to the institutional protocol for the evaluation of suspected febrile neutropenia in the pediatric ED, blood cultures are obtained from all available vascular access sites whenever technically feasible. This routinely includes at least two separate peripheral venipuncture sites and all indwelling central venous catheters present at the time of ED evaluation.

Neutropenia was defined as an absolute neutrophil count (ANC) < 1000/µL measured at or shortly after ED arrival. This threshold reflects the institutional clinical pathway for pediatric oncology patients with suspected febrile neutropenia and is routinely used in our center to trigger standardized evaluation and empiric antibiotic therapy. ANC values reported by the laboratory as undetectable were treated as zero for analysis.

Candidate predictors were selected a priori based on clinical relevance and routine availability at ED presentation. The transfusion-to-ED arrival interval was defined as the elapsed time in hours from the start of the most recent transfusion episode to the patient’s arrival in the ED. When multiple transfusions were administered within the 24-h window before presentation, the interval was calculated from the start time of the last transfused component, which we considered the most proximate transfusion exposure to the ED visit and a pragmatic way to handle overlapping transfusion episodes.

Body temperature was defined as the first documented temperature at ED triage, measured using standard digital thermometry, predominantly tympanic routes according to institutional protocol. C-reactive protein (CRP) was defined as the first serum concentration measured at ED presentation or within the first hour of ED care. Additional clinical and laboratory variables, including age, underlying diagnosis, transfused blood product type, and other routine chemistry and coagulation parameters, were recorded for descriptive and exploratory analyses.

### 2.3. Data Collection and Statistical Analysis

Data for all eligible encounters were extracted from the electronic medical record into an anonymized analysis dataset. Clinical, laboratory, and transfusion-related variables were collected according to the definitions described above. Continuous variables were summarized as medians with interquartile ranges (IQRs), and categorical variables as counts with percentages. For descriptive and univariable comparisons between encounters with and without BSI, continuous variables were compared using the Mann–Whitney U test, and categorical variables using the Chi-square test or Fisher’s exact test when expected cell counts were small. For multivariable model development, we focused on predictors that were routinely available at ED presentation (transfusion-to-ED arrival interval, temperature, ANC, and CRP). A complete-case approach was applied: encounters with missing values in any of these predictors or in the outcome (blood culture result) were excluded from the multivariable logistic regression and score derivation analyses. For descriptive tables, available data were used for each variable, and the extent of missingness for key variables is reported.

No formal a priori sample size calculation was performed; instead, all consecutive encounters that met the eligibility criteria during the study period were included. All statistical analyses and figure generation were performed using Python version 3.13 (Python Software Foundation) and standard scientific libraries.

### 2.4. Model Development and Score Derivation

The prediction model was developed using multivariable logistic regression with BSI as the dependent variable. Four predictors were specified in advance based on clinical plausibility and routine availability at ED presentation: transfusion-to-ED arrival interval, body temperature at triage, ANC, and CRP. These variables were entered into the model as continuous covariates. Encounters with missing values in any of these predictors or in the blood culture outcome were excluded from the multivariable analysis. We examined pairwise correlations among the candidate predictors and found no evidence of problematic collinearity (all variance inflation factors < 1.1). For each predictor, adjusted odds ratios (ORs) and 95% confidence intervals (CIs) were estimated, and overall model discrimination was assessed using the area under the receiver operating characteristic curve (AUC).

A simplified bedside score was then derived from the same four predictors. Each continuous predictor was converted to a binary indicator using prespecified cut-off values informed by clinical practice and inspection of receiver operating characteristic curves, selecting thresholds close to the maximum Youden’s index when feasible. Indicators were defined for higher temperature (≥38.5 °C), elevated CRP (≥2.5 mg/dL), profound neutropenia (ANC = 0/µL), and a longer transfusion-to-ED arrival interval (≥7 h). Integer point values were assigned according to the relative strength of association in the multivariable model: 2 points for ANC = 0/µL and 1 point each for higher temperature, elevated CRP, and a longer transfusion-to-ED interval, yielding a total score ranging from 0 to 5.

To relate the total score to the probability of BSI, a logistic regression model with the integer score as the single predictor was fitted in the study cohort. This model was used to estimate the predicted probability of BSI for each score value and to evaluate the diagnostic performance of selected score thresholds for risk stratification.

### 2.5. Performance Evaluation and Validation

For the multivariable logistic regression model, predicted probabilities of BSI were obtained for each encounter. Discrimination was assessed by constructing receiver operating characteristic (ROC) curves and calculating the AUC. The same approach was used to evaluate the discrimination of the simplified bedside score, treating the total score as a continuous predictor.

For the bedside score, diagnostic performance was further evaluated at selected integer cut-off values. At each threshold, sensitivity, specificity, positive predictive value, and negative predictive value were calculated, with particular attention to thresholds intended for ruling out BSI and for identifying higher-risk patients.

Internal validation was performed using bootstrap resampling. In each of 1000 bootstrap samples, the model was refitted and its performance was evaluated in the corresponding out-of-bootstrap observations to obtain optimism-corrected estimates of discrimination and calibration. Calibration for both the full model and the bedside score was assessed using calibration plots and Brier scores.

All performance measures were calculated in the same cohort used for model development. No separate external validation dataset was available.

### 2.6. Ethical Approval

This study was approved by the Institutional Review Board of Seoul National University Hospital, and the requirement for informed consent was waived because of its retrospective design.

## 3. Results

### 3.1. Study Population and Outcome Events

Of the 507 encounters screened for eligibility, 293 met the inclusion criteria for transfusion-associated febrile neutropenia. After excluding six encounters with incomplete data, 287 encounters were included in the final analysis (Figure 1). The median age of the cohort was 6.0 years (IQR, 3.0–11.0), and 153 encounters (53.3%) involved boys. The most common underlying diagnosis was a solid tumor (68.3%), followed by acute lymphoblastic leukemia (14.3%). The median transfusion-to-ED arrival interval was 7.6 h (IQR, 2.5–16.6 h). Patients were profoundly neutropenic at presentation, with a median ANC of 0/µL.

After applying the predefined adjudication criteria for contamination and colonization, BSI was identified in 39 of 287 encounters (13.6%), and the remaining 248 encounters (86.4%) were classified as non-BSI. Among the 39 BSI episodes, *Escherichia coli* and *Klebsiella pneumoniae* were the most frequently isolated pathogens.

During the study period, institutional protocols for the evaluation and management of pediatric febrile neutropenia in the ED, including transfusion and blood culture practices, remained essentially unchanged, indicating a stable clinical approach across the study years.

### 3.2. Univariable Predictors of BSI

In univariable analysis, several clinical and laboratory factors were associated with BSI. Encounters with BSI had a significantly longer median transfusion-to-ED arrival interval than non-BSI encounters (14.4 h [IQR, 8.2–19.8 h] vs. 5.6 h [2.4–16.3 h]; *p* = 0.002). The median body temperature at ED presentation was also higher in the BSI group (38.7 °C [38.2–39.5 °C] vs. 38.0 °C [37.5–38.4 °C]; *p* < 0.001).

Patients with BSI were more often profoundly neutropenic: an ANC of 0/µL was observed in 36 of 39 BSI encounters (92.3%) compared with 139 of 248 non-BSI encounters (56.0%; *p* < 0.001). C-reactive protein levels were also higher in BSI cases than in non-BSI cases (median, 2.7 mg/dL [1.2–3.9 mg/dL] vs. 1.2 mg/dL [0.5–2.5 mg/dL]; *p* < 0.001) (Table 1).

### 3.3. Multivariable Model and Score Development

**Figure 2:**
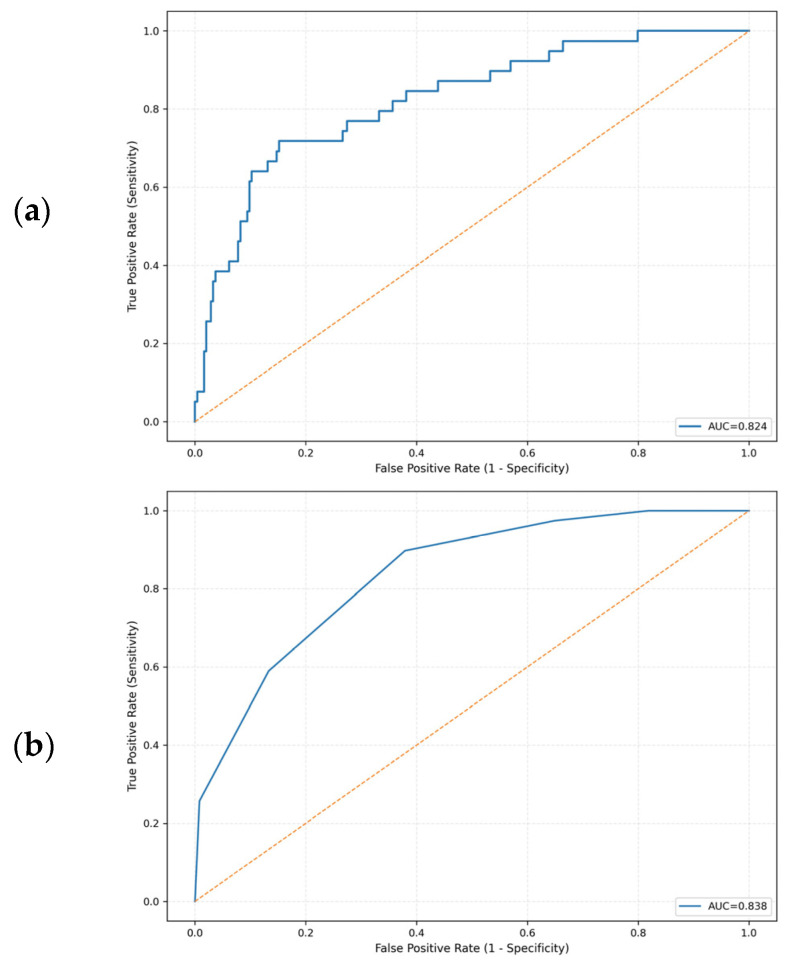
Receiver operating characteristic curves for predicting bloodstream infections. (**a**) Multivariable logistic regression model. (**b**) Simplified integer-based clinical score derived from the model. The yellow dashed line represents the reference line of no discrimination (AUC = 0.5).

In the multivariable logistic regression model including transfusion-to-ED arrival interval, body temperature at triage, ANC, and CRP as continuous predictors, higher temperature and a longer transfusion-to-ED arrival interval were associated with increased odds of BSI. The odds ratio (OR) for temperature was 2.62 (95% CI, 1.637–4.192), and the OR for the transfusion-to-ED arrival interval was 1.05 per 1-h increase (95% CI, 1.001–1.106). Lower ANC (OR, 0.98; 95% CI, 0.967–1.000) and higher CRP (OR, 1.10; 95% CI, 0.994–1.217) showed similar directions of association. The overall model demonstrated good discrimination, with an AUC of 0.82 (Table 2; Figure 2a).

On the basis of these four predictors, we derived a simplified integer-based clinical prediction score by applying prespecified cut-off values. The final score assigned 2 points for ANC = 0/µL and 1 point each for temperature ≥ 38.5 °C, CRP ≥ 2.5 mg/dL, and a transfusion-to-ED arrival interval ≥ 7 h, yielding a total score ranging from 0 to 5 points (Table 3).

### 3.4. Prediction Score Performance and Validation

The bedside clinical prediction score showed good discrimination, with an AUC of 0.84 (Figure 2b). At the prespecified lower-risk threshold, a score ≥ 2 correctly identified almost all BSI episodes, with a sensitivity of 97.4% and a negative predictive value (NPV) of 98.8%; only 1 of 39 BSI encounters was misclassified as low risk. At a higher threshold of ≥3, specificity increased to 59.7% while sensitivity remained 89.7%, with an NPV of 97.4%. In 1000 bootstrap resamples, the optimism-corrected AUC of the score was similar (0.83), indicating that the apparent discrimination was only minimally affected by overfitting.

## 4. Discussion

Our study of pediatric patients receiving chemotherapy who developed fever within 24 h after transfusion showed that four routinely available parameters at ED presentation—transfusion-to-ED arrival interval, body temperature, ANC, and CRP—can be combined into a simple bedside score to stratify BSI risk. In the multivariable model, higher temperature and a longer transfusion-to-ED arrival interval were associated with increased odds of culture-confirmed BSI, while lower ANC and higher CRP showed similar directions of association. The resulting 0–5 point score demonstrated good discrimination and strong rule-out performance in this cohort; at a threshold of ≥2 points, only 1 of 39 BSI encounters was classified as low risk. Because the score relies solely on information obtained during the initial ED evaluation, it has the potential to support more structured early risk assessment in children with post-transfusion febrile neutropenia.

Early and accurate risk stratification in pediatric FN is central to supportive care because BSI is a major driver of morbidity. Contemporary pediatric FN guidelines recommend prompt empiric antibiotics and the use of validated risk assessment tools to guide management [1]. Several pediatric FN risk tools (e.g., the SPOG 2003 adverse event score and the PICNICC model) have been developed and evaluated; however, their performance and calibration vary across settings, endpoints are heterogeneous, and some require data not readily available at ED presentation [7,8,10]. These models have substantially advanced risk-adapted care in pediatric oncology, but they were primarily designed for general episodes of febrile neutropenia rather than specifically for post-transfusion fever, and they do not explicitly incorporate transfusion-to-presentation timing, which is the focus of the present study.

We focused on a small core set of routinely available predictors at ED presentation. Temperature, ANC, and CRP are obtained as part of the initial evaluation in virtually all pediatric FN episodes in our setting and allow a rapid, objective appraisal of infection risk. These parameters are also among the most consistently reported predictors of adverse outcomes in pediatric FN, as shown in meta-analyses and biomarker reviews [8,11,12]. Although newer markers such as procalcitonin and interleukins (e.g., IL-6, IL-10) may provide additional information, they are not uniformly available and are less integrated into real-time ED workflows. In this context, we chose to base model development and score derivation only on temperature, ANC, CRP, and transfusion-to-presentation timing, aiming for a pragmatic tool that can be implemented in routine ED practice rather than an optimized model that depends on a wider array of less accessible predictors.

Recent transfusions can substantially complicate the clinical picture in children receiving chemotherapy. FNHTRs remain among the most common non-infectious reactions and typically occur during or within approximately 4 h after component administration, particularly with platelet and RBC transfusions. In this context, our finding that a longer transfusion-to-ED arrival interval is associated with BSI is biologically plausible. We hypothesize that this pattern reflects two partially overlapping processes: a rapid, passive inflammatory response to cytokines in the transfused product that characterizes FNHTR, and a slower, host-driven immune response to replicating pathogens that underlies true infection. At the same time, the observed association may also be influenced by behavioral and logistical factors in this retrospective cohort—for example, children who develop fever very soon after transfusion can only present with short intervals by definition, whereas those who remain febrile or unwell for several hours may arrive later. These mechanisms could contribute to the apparent risk gradient across the transfusion-to-presentation interval and underline the need for cautious interpretation and prospective evaluation of this predictor.

Interestingly, although previous hemovigilance data suggest that reaction profiles can vary by component [13,14], the transfusion type itself did not clearly stratify BSI risk in our cohort. Instead, the transfusion-to-ED arrival interval appeared to be a more informative predictor. Taken together, these observations suggest that, in children who develop fever after transfusion, episodes occurring outside the typical FNHTR time window may warrant greater clinical suspicion for BSI and closer diagnostic evaluation.

In developing the prediction model, we deliberately restricted the candidate predictors to four variables that are obtained in almost all episodes of pediatric febrile neutropenia at ED presentation in our setting and had very little missingness. This choice reflects a pragmatic emphasis on routine availability and simplicity rather than statistical optimization alone. ANC was prioritized over other hematologic indices such as hemoglobin or platelet count because it more directly reflects the depth of chemotherapy-induced myelosuppression and has consistently been associated with infection risk in prior FN studies. The decision to treat ANC = 0/µL as a separate category and to assign it greater weight in the score was informed by the univariable distribution in our cohort, in which the vast majority of BSI episodes occurred in encounters with an undetectable ANC, and by the clinical rationale that profound neutrophil depletion may mark a subgroup with particularly limited host defense.

Taken together, these clinical and practical considerations shaped the development of a focused, bedside-oriented prediction tool. Although not all four predictors retained strong independent associations with BSI at conventional significance thresholds in the multivariable model, their directions of effect were consistent with prior evidence and with the pathophysiology of infection in this population, and the overall model demonstrated good discrimination (AUC 0.82). The simplified 0–5 point score derived from these variables showed comparable performance (AUC 0.84) with close agreement between predicted and observed BSI risk. At the lower threshold of ≥2 points, the score achieved high sensitivity and a very high negative predictive value suitable for ruling out BSI, whereas a higher threshold of ≥3 points provided greater specificity for identifying higher-risk encounters. These operating characteristics are consistent with the principles of pediatric FN guidance, which combine prompt empiric antibiotics with risk-adapted management at presentation [1].

This study has several limitations. It was a retrospective, single-center analysis conducted in a specific pediatric oncology and transfusion practice environment, which may limit generalizability. The number of BSI events was modest, and all model development and performance assessment were conducted in the same cohort, so some overfitting and misclassification of BSI status remain possible despite predefined outcome criteria and bootstrap internal validation. In addition, routinely collected data did not allow us to fully reconstruct the clinical course (for example, initial diagnostic impressions, suspected infection foci, pre-ED antipyretic use, transfusion dosing, and downstream outcomes such as length of stay or mortality), which limits direct evaluation of how the score relates to subsequent illness severity and recovery. Finally, the model and score were derived in a single institutional setting with local laboratory reporting and practice patterns and have not yet been externally validated; prospective evaluation in independent pediatric cohorts, particularly in children with post-transfusion fever, will therefore be important before the score is used to guide routine clinical decisions.

In summary, among children receiving chemotherapy who develop post-transfusion fever, a simple 0–5 point bedside score based on temperature, ANC, CRP, and the transfusion-to-ED arrival interval provided useful early risk stratification for BSI in our single-center cohort. The score combined high negative predictive value at a lower threshold with greater specificity at a higher threshold, offering a straightforward framework for classifying encounters as lower or higher risk at ED presentation. Rather than directly changing management, our findings suggest that this approach could inform future risk-adapted pathways that aim to identify children who might safely be considered for less intensive strategies while maintaining vigilance for those at higher risk. Prospective multicenter validation and implementation studies should evaluate the clinical impact, antibiotic stewardship implications, and safety outcomes of incorporating this score into routine care.

## 5. Conclusions

In this single-center cohort of children receiving chemotherapy who presented with post-transfusion fever, a simple 0–5 point bedside score incorporating temperature, ANC, CRP, and the transfusion-to-ED arrival interval provided useful early stratification of bloodstream infection risk. The score combined high negative predictive value at a lower threshold with greater specificity at a higher threshold, offering a pragmatic framework for classifying encounters as lower- or higher-risk at emergency department presentation. Given the retrospective, single-center design of this study, prospective multicenter validation and prospective evaluation of its clinical utility are needed before the score can be incorporated into routine decision-making.

## Figures and Tables

**Table 1:** Univariable analysis of risk factors for bloodstream infection.

Variable		Non-BSI (*n* = 248)	BSI (*n* = 39)	*p* Value
Age, years		6.0 [3.0–11.0]	9.0 [3.0–13.0]	0.084
Sex		F: 114 (46.0%); M: 134 (54.0%)	F: 20 (51.3%); M: 19 (48.7%)	0.536
Transfusion-to-ED arrival, h		5.6 [2.4–16.3]	14.4 [8.2–19.8]	0.002
RBC only		110 (44.4%)	15 (38.5%)	0.603
PLT only		68 (27.4%)	13 (33.3%)	0.448
Both		68 (27.4%)	10 (25.6%)	1.000
Others		2 (0.8%)	1 (2.6%)	0.356
Temperature at triage, °C		38.0 [37.5–38.4]	38.7 [38.2–39.5]	<0.001
ANC,/µL		0.0 [0.0–91.0]	0.0 [0.0–0.0]	<0.001
Platelet, ×10^9^/L		46.0 [18.0–95.2]	21.0 [10.0–54.0]	0.002
Hemoglobin, g/dL		7.9 [7.1–9.0]	8.4 [7.2–9.1]	0.378
BUN, mg/dL		10.0 [8.0–12.0]	11.0 [9.0–13.5]	0.122
Creatinine, mg/dL		0.5 [0.4–0.5]	0.5 [0.4–0.6]	0.531
Total bilirubin, mg/dL		0.9 [0.7–1.2]	1.1 [0.9–1.4]	0.007
AST, U/L		26.0 [20.0–35.0]	24.0 [19.0–27.0]	0.098
ALT, U/L		30.0 [18.0–51.0]	29.0 [20.5–44.5]	0.675
INR ^†^		1.1 [1.1–1.2]	1.2 [1.1–1.3]	0.095
CRP, mg/dL		1.2 [0.5–2.5]	2.7 [1.2–3.9]	<0.001
Procalcitonin, ng/mL ^‡^		0.2 [0.1–0.4]	0.2 [0.2–1.2]	0.549
Indwelling CVC present		159 (64.1%)	33 (84.6%)	0.011
Diagnosis				
ALL		36 (14.5%)	5 (12.8%)	1.000
AML		12 (4.8%)	5 (12.8%)	0.064
Lymphoma		17 (6.9%)	8 (20.5%)	0.011
Solid		175 (70.6%)	21 (53.8%)	0.043
Pathogen (BSI only)	*Escherichia coli* 11 (28.2%); *Klebsiella pneumoniae* ssp. *pneumoniae* 11 (28.2%); *Enterobacter cloacae* complex 8 (20.5%); Viridans group *Streptococcus* 5 (12.8%); *Pseudomonas aeruginosa* 2 (5.1%); *Bacillus cereus* group 1 (2.6%); *Streptococcus mitis* 1 (2.6%)			

Values are median [IQR] or %; *p* values from Mann–Whitney U (continuous) or χ^2^/Fisher’s exact test (categorical); ^†^ INR available in 58 patients in the Non-BSI group and 17 in the BSI group; ^‡^ Procalcitonin available in 32 patients in the Non-BSI group and 5 in the BSI group; Unless otherwise indicated, *n* = 248 for Non-BSI and *n* = 39 for BSI. ANC, absolute neutrophil count; ALT, alanine aminotransferase; AST, aspartate aminotransferase; BSI, bloodstream infection; BUN, blood urea nitrogen; CVC, central venous catheter; CRP, C-reactive protein; INR, international normalized ratio; PLT, platelet; RBC, red blood cell; ED, emergency department; ALL, acute lymphoblastic leukemia; AML, acute myeloid leukemia. Bacterial species are shown in italics.

**Table 2:** Multivariable logistic regression for bloodstream infection.

	Adjusted OR (95% CI)
Transfusion-to-ED arrival (per h)	1.05 (1.001–1.106)
Temperature at triage (per 1 °C)	2.62 (1.637–4.192)
C-reactive protein, mg/dL (per 1)	1.10 (0.994–1.217)
Absolute neutrophil count/µL (per 1)	0.98 (0.967–1.000)

ED, Emergency department; OR, Odds ratio; CI, Confidence interval.

**Table 3:** Simplified integer-based clinical prediction score.

Predictor	Dichotomized Rule	Points
Temperature at triage	≥38.5 °C	1
C-reactive protein	≥2.5 mg/dL	1
Absolute neutrophil count	=0/µL	2
Transfusion-to-ED arrival	≥7 h	1

ED, Emergency department; total possible score ranges from 0 to 5 points.

## Data Availability

The data presented in this study are available on reasonable request from the corresponding author. The data are not publicly available due to privacy and ethical restrictions.

## Abbreviations

The following abbreviations are used in this manuscript:

FN	Febrile Neutropenia
BSI	Bloodstream Infection
ED	Emergency Department
ANC	Absolute Neutrophil Count
CRP	C-reactive Protein
FNHTR	Febrile Non-Hemolytic Transfusion Reaction
